# Determinants of post-discharge stunting among diarrhoeal children aged 2–23 months in Bangladesh: findings from Antibiotics for Children with Severe Diarrhea (ABCD) trial

**DOI:** 10.7189/jogh.15.04185

**Published:** 2025-06-27

**Authors:** Sharika Nuzhat, Rina Das, Md. Farhad Kabir, Md. Ahshanul Haque, Abu Sadat Mohammad Sayeem Bin Shahid, Mehnaz Kamal, Md. Tanveer Faruk, Tahmeed Ahmed, Mohammod Jobayer Chisti

**Affiliations:** 1 Clinical and Diagnostic Services, icddr,b, Dhaka, Bangladesh; 2 Artificial Intelligence and Cyber Futures Institute, Charles Sturt University, New South Wales, Australia; 3 Nutrition Research Division, icddr,b, Dhaka, Bangladesh; 4 Gangarosa Department of Environmental Health, Rollins School of Public Health, Emory University, Atlanta, Georgia, USA; 5 James P. Grant School of Public Health, BRAC University, Dhaka, Bangladesh; 6 Department of Global Health, University of Washington, Seattle, Washington, USA

## Abstract

**Background:**

Despite the global burden of stunting, data regarding the determinants of stunting observed on follow-up after discharge from health care facilities among children treated for severe diarrhoea are limited. We investigated factors influencing stunting during post-discharge follow-up among under-2 children treated for severe diarrhoea. We developed a predictive model to estimate stunting prevalence and identify risk factors without post-discharge anthropometry.

**Methods:**

We analysed data from two sites in Bangladesh participating in the multi-country, double-blind, randomised clinical trial on Antibiotics for Children with Severe Diarrhoea among children aged 2–23 months, from 2017 to 2019. Severe diarrhoea was defined as a child who had acute diarrhoea with severe/some dehydration, or moderate wasting, or severe stunting on admission. Multiple linear regression was constructed to predict the independent factors associated with stunting among the enrolled children who were followed for 180 days after hospital discharge. We developed a predictive model for stunting using 75% of the available data for training, with the remaining 25% reserved as a test set to evaluate model performance. We then applied this predictive model to the children not enrolled in the trial but admitted to the hospital for diarrhoea to estimate the predictive prevalence and determinants of post-discharge stunting at 180 days.

**Results:**

Of the 1431 enrolled children, 589 (41.2%) were stunted at enrolment. By day 180, stunting increased to 698 (49.3%). Linear growth of diarrhoeal children over this follow-up period was positively associated with enrolment length-for-age z score (LAZ), maternal body mass index (BMI), maternal education, duration of diarrhoea, and breastfeeding. We applied the predictive model developed on the enrolled patients' data to the 33 341 children admitted for diarrhoea but not enrolled in the trial. The model estimated that 6142 children (18.4%) in this non-follow-up group would have been stunted at 180 days. Persistent stunting at 180 days for these not-followed children was predicted to be positively associated with dehydration on admission and a higher number of children in the household, while a negative association was predicted with formal parental education and increased maternal BMI.

**Conclusions:**

Our study highlights the need for targeted interventions to mitigate persistent stunting in this vulnerable group recovering from diarrhoea.

Diarrhoeal diseases and impaired child growth are major global public health problems [1], affecting children in low-resource settings. Malnutrition in children under five is measured using stunting, wasting, and underweight. These indicators help to show the scale and pattern of undernutrition. This is aligned with monitoring progress toward the nutritional targets under Sustainable Development Goal 2 to end hunger and all forms of malnutrition by 2030. In 2023, United Nations Children's Fund (UNICEF)/World Health Organization (WHO)/World Bank Group Joint Child Malnutrition Estimates reported that globally in 2022, stunting affected 22.3%, or 148.1 million, children under five [2,3]. While global stunting has declined since 2000, accelerated progress is needed to halve stunting by 2030. At current rates, the world is on track to miss these 2030 goals by 39.5 million children [2]. Various public health programmes have been implemented worldwide over the last two decades, aiming to curb stunting prevalence. In some geographic settings, the reduction goal remains unfulfilled, underscoring the persisting challenge. Available evidence from UNICEF/WHO/World Bank Group Joint Child Malnutrition Estimates in 2023 indicates South Asia bears a disproportionate burden of childhood stunting, with approximately 37% of under-5 children estimated to be stunted in the region [2]. The prevalence of stunting among under-5 Bangladeshi children significantly declined from 49.8% in 2004 to 30.7% in 2017 − 18, according to data from Bangladesh Demographic and Health Surveys (BDHS) [4], indicating notable progress in reducing chronic undernutrition over this time. Still, Bangladesh has yet to reach the 15% threshold for stunting, defined as low prevalence and an end to an emergency nutritional situation [5].

Childhood diarrhoeal disease is a major driver of undernutrition in low-resource settings. Diarrhoea contributes to environmental enteric dysfunction (EED), characterised by intestinal inflammation and morphological changes impairing nutrient absorption [6]. This promotes bacterial translocation, immune activation, and chronic gut inflammation, raising energy expenditure and suppressing growth via cytokines [7]. Both diarrhoea and EED-induced dysfunction compromise children's nutrition and increase stunting risk. Stunting has been linked to numerous adverse health and developmental consequences, including increased mortality, heightened morbidity, and impairments in cognitive and motor functioning [8].

Acute diarrhoea is a major cause of childhood morbidity and mortality in LMICs and frequently leads to impairments in linear growth even after clinical recovery [9]. However, post-discharge follow-up and monitoring of nutritional status are often lacking in resource-poor areas. Persistent stunting following discharge after treatment of acute diarrhoea in children who presented with stunting on admission indicates a failure to recover lost growth potential [10]. Identifying modifiable risk factors could guide interventions to prevent long-term consequences.

We aimed to address evidence gaps in predicting post-discharge stunting among children under two years treated for acute diarrhoea in health care facilities. While associations between diarrhoeal illness, EED, and impaired growth are well established, few studies have examined post-discharge stunting risk, especially in low-resource settings where follow-up anthropometry is often infeasible.

Our objectives were to 1) identify clinical, nutritional, and sociodemographic determinants of stunting 180 days post-discharge, and 2) to develop and validate a predictive model to estimate stunting risk in the absence of follow-up data. We hypothesised that specific baseline characteristics at admission would be independently associated with subsequent stunting and could inform support targeted follow-up strategies.

Existing literature largely focuses on community-based cohorts or overall malnutrition, with limited attention to post-discharge growth trajectories. By leveraging cohort data from Bangladesh, we address this gap with a novel predictive approach. While the parent study (ABCD trial) [11] described population-level trends in undernutrition, our analysis focuses on individual-level risk prediction.

This model could help prioritise vulnerable children for follow-up in resource-limited settings, supporting more efficient health care resource allocation. To our knowledge, this is the first predictive tool developed for post-discharge stunting in this context, with the potential to generate targeted interventions to improve child health.

## METHODS

### Study design and setting

The multi-country Antibiotic for Children with Severe Diarrhea (ABCD) trial was a double-blind, randomised controlled trial that enrolled high-risk children aged 2–23 months presenting with acute non-bloody diarrhoea from 36 sites across Bangladesh, India, Kenya, Malawi, Mali, Pakistan, and Tanzania between 2017 and 2019 [11,12]. Participants were recruited from both hospital outpatient departments and community health centres in urban and rural settings. We extracted the related data from the ABCD trial database from sites located in the urban Dhaka Hospital and Mirpur Treatment Centre of the International Centre for Diarrheal Disease Research, Bangladesh (icddr,b) in Dhaka, Bangladesh. The icddr,b Dhaka Hospital is the world's largest diarrhoeal disease hospital, treating an average of 350 patients daily, and the icddr,b Mirpur Treatment Centre is a 50-bed hospital that has treated diarrhoeal patients for 10 years. Both sites have been previously described [13,14].

### Study participants

#### Screening and recruitment

The study participants were children aged 2–23 months presenting with acute severe diarrhoea to participating health care facilities. Acute severe diarrhoea was defined if a child with acute watery diarrhoea (≥3 watery stools in the previous 24 hours) had any one of the following criteria: the presence of some or severe dehydration; moderate wasting defined as a weight-for-length z-score of>−3 and≤−2 or a mid-upper arm circumference (MUAC) of ≥115 mm (mm) and <125 mm; or severe stunting (length-for-age z score (LAZ) is<−3 standard deviation (SD) from the median of the WHO child growth standards). Children were excluded if they had dysentery (blood in stool) or suspected cholera requiring antibiotic treatment; severe acute malnutrition; signs of other infections necessitating antibiotics; receipt of antibiotics in the prior 14 days; previous enrolment in another clinical trial; or residence outside the study area. More details on inclusion and exclusion criteria are provided in the trial protocol [11].

### Study procedures

The design and methodology of the main trial have already been detailed previously [11]. All children 2–23 months of age presenting with acute severe diarrhoea admitted to the Bangladesh sites were screened for eligibility using a standardised form [11]. Children presenting with some or severe dehydration were monitored during initial stabilisation, where oral and/or intravenous rehydration was administered according to WHO guidelines [15]. Any acute medical needs were treated according to the standard of care. Once rehydrated and clinically stable, eligible participants were formally enrolled in the study [11]. Enrolled children were followed up in the hospital on days 90 and 180 to determine their vital status, hospitalisations, health status, and anthropometry (weight, length/height, and MUAC). All children received standard care for diarrhoeal disease, including zinc, rehydration, and nutritional counselling, following WHO guidelines [15] in the hospital, regardless of enrolment in the trial [11]. We included all enrolled and screened-out participants for our analysis.

In addition to the enrolled cohort, we included data from children screened for eligibility but not enrolled in the main ABCD trial (‘screened-out’ participants). According to WHO guidelines, these children did not meet one or more inclusion criteria to enrol in the ABCD trial but received standard care for diarrhoeal illness. As follow-up data regarding outcome were not collected for this group (‘screened-out’ participants) as per the ABCD trial protocol, they were not considered lost to follow-up. Therefore, missing data mechanisms and imputation strategies were not applicable here. Predictions generated from the final logistic regression model were applied to this screened population to estimate the potential burden of post-discharge stunting, assuming comparable baseline care and disease severity at presentation. As no follow-up anthropometry was collected for this group, extrapolation assumes that model predictors retain similar associations in this population. While this approach provides insight into the potential broader burden of post-discharge stunting, we acknowledge that predictive uncertainty may be greater in this non-followed population. The absence of outcome data precluded formal model calibration or bias adjustment, and findings should therefore be interpreted as exploratory and hypothesis-generating.

### Study outcome

For our study, the primary outcome was a change in linear growth, which was measured as a change in the length/height-for-age z score (L/HAZ) between enrolment and day 180 after enrolment among the enrolled children. Forecasting of linear growth was also calculated among the children who were not enrolled in the trial. The Z-score scale = (observed value − average value of the reference population)/standard deviation value of the reference population was used to calculate length-for-age (LAZ), weight-for-age (WAZ), and weight-for-length (WLZ) z scores as indicators of child nutritional status according to the 2006 WHO Standards for Children [16].

### Predictor variables

**Figure 1:**
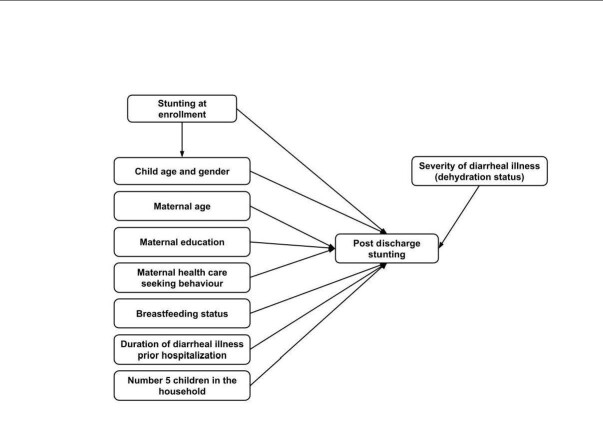
Factors associated with stunting at enrolment and post-discharge follow-up at days 180.

Based on a comprehensive literature review, previous descriptive studies, and data availability in our investigation, the independent variables were considered explanatory variables. The predictor variables are child age, gender, characteristics of diarrhoea, antibiotic use before hospitalisation, breastfeeding status, parental education, maternal age, maternal body mass index (BMI), number of children under five in the household, dehydration status during hospital admission, and duration of hospitalisation. More details were described elsewhere [13]. **Figure 1** shows the factors associated with stunting at enrolment and post-discharge follow-up at days 180.

### Data analysis and quality control

The ABCD trial team collected and managed the data using REDCap electronic data capture tools hosted at each participating site of the ABCD trial [12]. Only site investigators and data managers had access to ongoing de-identified data downloads and summary statistics reports, including frequencies of missing data, to facilitate data cleaning and validation. Participant data were securely stored locally at each study site, including Bangladesh, by institutional guidelines and regulations.

We conducted descriptive analyses to characterise the study population at baseline. Categorical variables were summarised using frequencies and proportions, while continuous variables were reported as means with standard deviations (SDs). Comparisons between stunted and non-stunted children at 180-day follow-up were performed using χ^2^ tests for categorical variables and independent *t* tests for continuous variables, where appropriate. These descriptive comparisons provided context for identifying potential risk factors to inform multivariable modelling.

To identify independent predictors of linear growth at 180 days post-discharge, we employed a multivariable linear mixed-effects model with child-specific random intercepts to account for repeated measures of length-for-age z-scores (LAZ) at enrolment (day 1), post-discharge (day 90, and day 180). This model allowed for the correction of within-child correlation due to the longitudinal nature of the data. Fixed effects included baseline demographic, clinical, anthropometric, and socioeconomic variables, selected a priori based on clinical relevance and statistical significance (*P* < 0.25) in bivariate analyses. Time-varying exposures were also included as fixed effects. Potential predictors considered were demographic (age, gender, socioeconomic status), clinical (acute diarrhoea severity, co-morbidities), and nutritional factors (baseline anthropometric measures, feeding practices). We ensured that selected variables exhibited sufficient variability and were not influenced by measurement error.

Predictors were identified through a directed acyclic (DAG) approach (Figure S1 in the **Online Supplementary Document**) based on prior knowledge of the relationships between predictors and the outcome (stunting). We included potential confounders (*e.g*. baseline nutritional status, age, gender) in the regression model to avoid bias. Additionally, interaction terms between key covariates (*e.g*. age and nutritional status) were tested to assess effect modification, and non-significant interaction terms were excluded from the final model.

To estimate stunting risk at 180 days (binary outcome), we derived predicted LAZ values from the mixed-effects model and classified children as stunted or not using the WHO threshold (LAZ<−2). Model performance was assessed by comparing predicted stunting classifications against observed outcomes, using sensitivity, specificity, and overall accuracy. We employed a train-test split approach, with 75% of the data used for model development (training set) and 25% for validation (test set). The model's predictive power was evaluated using these performance metrics in the test data set.

A multivariable logistic regression model was then developed using the training data set to identify factors independently associated with post-discharge stunting. Variable selection was guided by both clinical relevance and statistical significance in bivariate screening. For model assumptions and selection, we evaluated the appropriateness of the logistic regression model by checking for linearity in the log-odds of the predictors, using graphical methods (*e.g*. boxplots and scatter plots). Multicollinearity was assessed using the variance inflation factor (VIF); no included variables exceeded a VIF threshold of 5. The final model’s predictive performance was evaluated in the held-out test data set. Statistical significance was defined as *P* < 0.05 for all hypothesis tests. We have included 95% confidence intervals (CIs) for all key effect estimates. Additionally, we report effect sizes (*e.g*. odds ratios for logistic regression) alongside their corresponding CIs, which better convey the magnitude of the observed associations and the precision of our estimates. All analyses were conducted using Stata, version 17 (StataCorp, College Station, TX, USA). The directed acyclic graph DAG file was created by RStudio 2024.12.1.

### Ethical approval

The WHO Ethics Review Committee approved the ABCD trial, and icddr, b Ethics Committee comprised the Research Review and Ethical Review Committee. Written informed consent was obtained from parents/caregivers of enrolled children, and verbal consent from parents of screened children, following relevant ethical norms and regulations. The study followed the CONSORT reporting guideline for randomised controlled trials [11].

## RESULTS

### Baseline characteristics of the enrolled children

**Figure 2:**
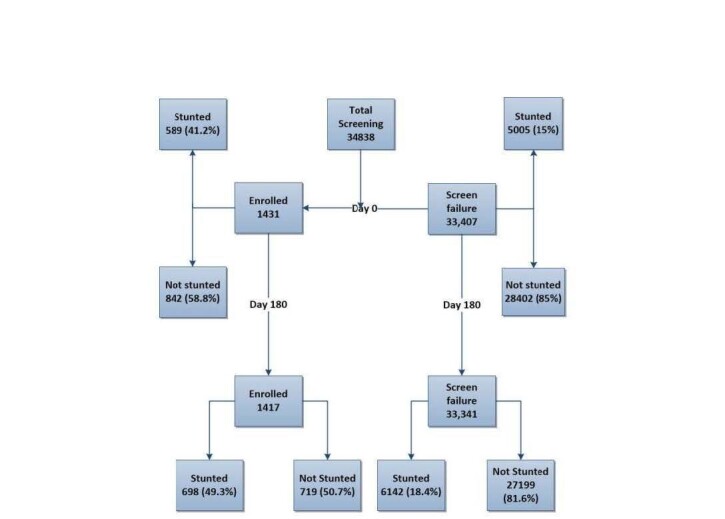
Selection of study participants and prevalence of post-discharge stunting among enrolled and screened failures.

A total of 34 838 children screened for eligibility in Bangladesh were included in our analysis. Of these, 1431 enrolled in the ABCD trial and provided anthropometric measures at enrolment (day 1), day 90, and day 180 follow-ups (**Figure 2**). Table S1 in the **Online Supplementary Document** shows the baseline characteristics of all screened children of the ABCD trial at the Bangladesh site. The percentage of stunting was higher among 2-to-11-month-old children among the enrolled and non-enrolled cases. The history of prior antibiotic consumption in the last 14 days was higher in non-stunted children compared to stunted children (40.2 *vs*. 33.4%). The percentage of maternal (7.1 *vs*. 14.3%) and paternal illiteracy (8.9 *vs*. 16.8%) was lower in non-stunted children than in stunted children among all screened cases. The median maternal BMI was comparatively lower in mothers of stunted children compared to non-stunted children among all screened children (22.75 *vs*. 23.92). The median duration of hospitalisation was also less stunted in cases (2.67 days *vs*. 4.03 days) among all screened children.

### Predictive prevalence of stunting among those without a post-discharge anthropometric assessment

At enrolment, 589 (41.2%) enrolled children were stunted. The prevalence of post-discharge stunting increased to 698 (49.3%) among these children at day 180. The remaining 33 407 children were not enrolled in the ABCD trial but were admitted to the hospital for treatment of acute diarrhoea. Among these non-enrolled children, 5005 (15.0%) were stunted based on screening anthropometry on day 1. A multiple logistic regression model predicting stunting at day 180 among these non-enrolled children estimated that 6142 (18.4%) would be stunted (**Table 1**).

**Table 1:** Predictive prevalence of stunting among those without a post-discharge anthropometric assessment

Nutritional status	Day 1		Day 180 original		Day 180 predicted	
**Stunting**	**Enrolled, n (%)**	Screened failure*, n (%)	**Enrolled, n (%)**	Screened failure*, n (%)	**Enrolled, n (%)**	Screened failure*, n (%)
Stunted	589 (41.2)	5005 (15.0)	698 (49.3)	N/A†	671 (47.0)	6142 (18.4)
Not stunted	842 (58.8)	28 402 (85.0)	719 (50.7)	N/A†	756 (53.0)	27 199 (81.6)

*Not enrolled in the ABCD trial but treated in the hospital for acute diarrhoea.

†Screen failure children (not enrolled) were not followed up.

**Figure 3:**
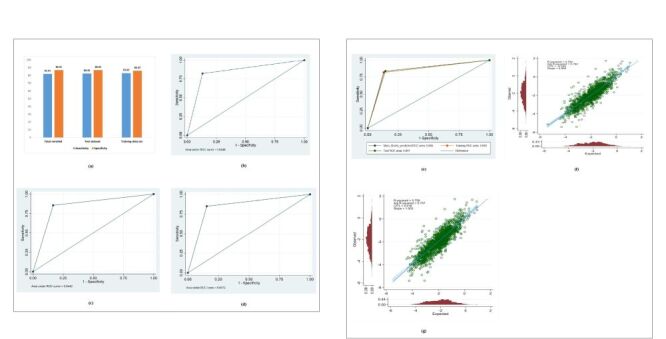
**Panel A.** Predictive performance of the model. **Panel B.** Post-estimation ROC and AUC for the entire model (after running the linear prediction model). **Panel C.** Post-estimation ROC and AUC for the model used 75% data as training (after running the linear prediction model). **Panel D.** Post-estimation ROC (Receiver Operating Characteristic) curve and AUC (Area Under the Curve) for the model used 25% data as test (after running the linear prediction model). **Panel E.** Post-estimation ROC and AUC for all the models used. **Panel F.** Calibration plot (training data set). **Panel G.** Calibration plot (test data set). Both Calibration plots show the calibration curves very close to the 45° line. CITL = 0.005 (training). The model is extremely well-calibrated. No action required. CITL = 0.018 (test). Suggests marginally more systematic error, but is still excellent. Prioritise recalibration only if the context requires perfection (*e.g*. clinical decision-making).

To check the validation, data were randomly divided into a training data set (75% of the data) and a test data set (25%), for calculating the algorithms' accuracy (**Figure 3**). The same training and test sets were used for all algorithms and prediction performance. **Figure 3**, Panels A–G provides vital performance metrics for evaluating the various algorithms, specifically their sensitivity and specificity in identifying stunting. The displayed algorithm achieved a notably high sensitivity of 82.45%, indicating strong potential to minimise missed stunting diagnoses. A specificity of 86.91% for the algorithm signifies good accuracy in discerning non-stunting children and limiting incorrect positive diagnoses. Together, these key metrics allow a direct comparison of how proficient each algorithm proves in distinguishing stunting *vs*. non-stunting children.

The models' performance was evaluated using Area Under the Curve (AUC), which was 0.8446 for the overall model (**Figure 3**). The AUC value for the training and test data sets was also assessed separately, showing consistent and acceptable accuracy (training data = 0.8442; test data = 0.8472). Calibration plots for both training and test data sets demonstrated that the predicted outcomes closely aligned with observed outcomes, with curves lying near the 45° line (**Figure 3**). These indicate good model calibration and suggest minimal overfitting.

### Factors associated with linear growth (LAZ/HAZ) in day 180 follow-up among enrolled children

In the multiple linear regression model (**Table 2**), linear growth of diarrhoeal children after 180 days of enrolment has a positive association with enrolment LAZ (coeff. = 0.82, 95% confidence interval (CI) = 0.8, 0.85, *P* < 0.001), maternal BMI (coeff. = 0.01, 95% CI = 0.002, 0.02, *P* = 0.011), maternal primary education (coeff. = 0.09, 95% CI = 0.01, 0.17, *P* = 0.028), diarrhoeal duration (coeff. = 0.02, 95% CI = 0.0, 0.03, *P* = 0.053). The presence of children under five in the family had a significant negative association with linear growth on day 180 (coeff. = −0.12, 95% CI = −0.19, 0.05, *P* = 0.001).

**Table 2:** Factors associated with linear growth (LAZ/HAZ) in day 180 follow-up among 2–23 months-old children in Bangladesh: results of multiple linear regression modeling (dependent variable – LAZ/HAZ) of enrolled children (n = 1431)

Variables	Unadjusted		Adjusted*	
	**Coeff. (95% CI)**	***P* value**	**Coeff. (95% CI)**	***P*-value**
Age in months				
*2–11*	Ref.		Ref.	
*12–23*	−0.27 (−0.39, −0.16)	<0.001	−0.30 (−0.40, −0.19)	<0.001
Gender (male)	−0.42 (−0.54, −0.31)	<0.001	−0.39 (−0.50, −0.28)	<0.001
Maternal BMI	0.03 (0.01, 0.04)	<0.001	0.01 (0, 0.03)	0.061
Maternal age	0.01 (0.00, 0.02)	0.014	0.02 (0.01, 0.03)	0.002
Maternal education				
*No formal education*	Ref.		Ref.	
*Below primary*	0.08 (−0.12, 0.28)	0.440	0.04 (−0.15, 0.23)	0.704
*Primary and above*	0.33 (0.18, 0.48)	<0.001	0.34 (0.19, 0.49)	<0.001
Paternal education				
*No formal education*	Ref.		Ref.	
*Below primary*	0.04 (−0.18, 0.25)	0.733	0.06 (−0.14, 0.26)	0.572
*Primary and above*	0.24 (0.09, 0.38)	0.001	0.17 (0.03, 0.31)	0.019
Under 5 children at a household	−0.13 (−0.27, 0.01)	0.065	−0.12 (−0.25, 0.00)	0.059
Maternal decision-making behaviour on health care seeking				
*No*	Ref.		Ref.	
*Yes*	0.10 (−0.04, 0.25)	0.166	0.09 (−0.05, 0.22)	0.226
Duration of diarrhoea before hospitalisation (days)	−0.004 (−0.04, 0.03)	0.803	0.02 (−0.02, 0.05)	0.366
Dehydration status				
*No dehydration*	Ref.		Ref.	
*Some/severe signs of dehydration*	0.65 (0.52, 0.77)	<0.001	0.68 (0.55, 0.81)	<0.001
Breastfeeding status				
*Exclusive breastfeeding*	Ref.		Ref.	
*Mixed*	−0.12 (−0.62, 0.37)	0.625	0.04 (−0.42, 0.5)	0.857
*Non-breastfed*	−0.15 (−0.70, 0.39)	0.586	−0.12(−0.63, 0.39)	0.647
Number of loose stools within 24 h	0.02 (−0.001, 0.03)	0.007	−0.001 − 0.01, 0.01)	0.821

CI – confidence interval, Coeff. – coefficient, LAZ – length-for-age

*Outcome variable: LAZ/HAZ at day 180 follow-up after enrollment; adjusted for age, sex, breastfeeding status, parental education, enrolment LAZ/HAZ, maternal decision-making ability on health care seeking, maternal age and BMI, number of under-five children at household, duration of diarrhoea before hospital admission, number of loose stools in last 24 h; the presence of some/severe dehydration and having under 5 children at home.

### Factors associated with stunting among children who were not enrolled on day 1 and predicted post-discharge stunting on day 180

**Table 3** displays the associated factors among the stunted children who were not enrolled. Older children (adjusted odds ratio (aOR) = 1.36; 95% CI = 1.28, 1.45) and male children (aOR = 1.47; 95% CI = 1.38, 1.57) were more likely to be associated with stunting. After adjusting age, gender, breastfeeding status, parental education, enrolment WAZ, maternal decision-making ability on health care seeking, maternal age and BMI, number of under-five children at household, duration of diarrhoea before hospital admission, number of loose stools in last 24 hours; the children presented with some/severe dehydration (aOR = 1.61; 95% CI = 1.31, 1.97) at enrolment and having increased number of under five children at household (aOR = 1.2; 95% CI = 1.12, 1.29) were more likely to be associated with stunting at day 1. Compared to no parental formal education, if the parents' education level is primary and above, it reduces the risk of stunting among the under two who were treated for acute diarrhoea. An increase in maternal age (aOR = 0.98; 95% CI = 0.97, 0.99) and an increase in maternal BMI (aOR = 0.97; 95% CI = 0.96,0.97) have a protective effect on childhood stunting on day 1. Surprisingly, the duration of diarrhoea, the number of loose stools within the first 24 hours, breastfeeding, and maternal decision-making regarding health care seeking were not associated with childhood stunting on day 1.

**Table 3:** Associated factors of children's stunting status who were not enrolled on Day 1 but treated for watery diarrhoea in the hospital and predicted post-discharge stunting on Day 180 (n = 33 341)*

Variables	On admission		Day 180 (post-discharge)	
**aOR (95% CI)**	***P*-value**	**aOR (95% CI)**	***P*-value**
Age in months				
*2–11*	Ref			
*12–23*	1.36 (1.28, 1.45)	<0.001	1.28 (1.21, 1.36)	<0.001
Gender (male)	1.47 (1.38, 1.57)	<0.001	1.47 (1.38, 1.56)	<0.001
Dehydration status				
*No dehydration*	Ref			
*Some/severe dehydration*	1.61 (1.31, 1.97)	<0.001	1.59 (1.31, 1.93)	<0.001
Breastfeeding status				
*Exclusive breastfeeding*	Ref			
*Mixed*	0.88 (0.7, 1.11)	0.285	0.51 (0.42, 0.62)	<0.001
*Non-breastfed*	1.15 (0.88, 1.48)	0.303	0.49 (0.39, 0.62)	<0.001
Maternal decision-making on health care seeking				
*No*	Ref			
*Yes*	0.98 (0.9, 1.06)	0.602	1.03 (0.96, 1.12)	0.385
Maternal BMI	0.97 (0.96, 0.97)	<0.001	0.95 (0.94, 0.95)	<0.001
Maternal age	0.98 (0.97, 0.99)	<0.001	0.97 (0.97, 0.98)	<0.001
Maternal education				
*No formal education*	Ref			
*Below primary*	0.79 (0.69,0.91)	0.001	0.83 (0.73, 0.94)	0.004
*Primary and above*	0.59 (0.53,0.66)	<0.001	0.52 (0.47, 0.57)	<0.001
Paternal education				
*No formal education*	Ref			
*Below primary*	0.95 (0.82, 1.1)	0.486	0.96 (0.85, 1.10)	0.590
*Primary and above*	0.61 (0.55, 0.67)	<0.001	0.49 (0.45, 0.54)	<0.001
Under five children in the household	1.2 (1.12, 1.29)	<0.001	1.49 (1.39, 1.58)	<0.001
Duration of diarrhoea before hostpitalisation (days)	1.01 (1.0, 1.02)	0.129	0.98 (0.97, 0.99)	0.002
Number of loose stools within 24 h	1.0 (0.99, 1.01)	0.764	1.01 (1.00, 1.02)	0.027

aOR – adjusted odds ratio, BMI – body mass index, CI – confidence interval

*Adjusted for age, gender, breastfeeding status, parental education, enrollment LAZ/HAZ, maternal decision-making ability on health care seeking, maternal age and BMI, number of under-five children in household, duration of diarrhoea before hospital admission, and number of loose stools in last 24 h. Stunting was predicted based on the enrolled children.

At Day 180 on post-discharge follow-up, in **Table 3**, the predictive model shows post-discharge stunting among not enrolled children (n = 33 341) has been associated with admission age (aOR = 1.28; 95% CI = 1.21, 1.36), male gender (aOR = 1.47; 95% CI = 1.38, 1.56), having some/severe dehydration at day 1 (aOR = 1.59; 95% CI = 1.31,1.93), and the number of children under five at home. An upper level of education can reduce persistent stunting. An increase in maternal age and BMI can reduce the persistence of stunting on day 180. On the contrary, exclusive breastfeeding has no protective effect on stunting. However, stunting on day 180 was associated with a slightly shorter average duration of diarrhoea in days and suggested a small increase in the odds of more loose stools.

## DISCUSSION

Our analysis aimed to identify risk factors for stunting among children aged 2–23 months and to predict post-discharge stunting among children under two who failed to reach our follow-up process. Our most important observation is the significant relationship between post-discharge stunting in the next 180 days and several factors: low parental education, low maternal BMI, and the presence of two or more under-five children in households. Additionally, some/severe dehydration on admission due to diarrhoea is associated with post-discharge stunting. Interestingly, exclusive breastfeeding was not found to be associated with post-discharge stunting.

We identified factors (using on-admission data) associated with stunting among children aged 2–23 months with diarrhoea who were followed in the Bangladesh site of the ABCD trial. We also used on-admission information of screened patients who were not enrolled to get the association of these factors with stunting. We predicted these factors' association with post-discharge stunting at day 180 follow-up. The predictive model demonstrated reasonably good accuracy in predicting stunting risk among children who were enrolled, with a sensitivity of 82.01% and a specificity of 86.9%. Due to a lack of reliable data, nutrition-specific data in national survey data sets such as Bangladesh Demographic and Health Surveys and UNICEF’s Multiple Indicator Cluster Survey, key determinants of stunting, such as food consumption and birth outcomes *etc*. were not well represented.

Despite these limitations, maternal characteristics including parity, birth spacing, and height of the mother, showed modest predictive value for linear growth retardation and stunting across the population analysed. These characteristics are often used as markers of both the past and future risk of linear growth retardation. An area with a high prevalence of childhood stunting reflects that children are developing in an environment suboptimal for growth and prevents children from reaching their genetic height potential. This has a consequence, as it can limit an individual's potential and hinder society's ability to achieve sustained economic development and prosperity.

Evidence demonstrates that the period from conception to 24 months is critical for linear growth, with rapid deceleration thereafter [17]. This observation led to assertions that interventions beyond 1000 days result in negligible height gains [18]. However, while the window for catch-up growth may narrow, evidence suggests that cognitive domains like reasoning and problem-solving continue to develop throughout adolescence [19]. Recent data have not established effective interventions in post-24-month programming nor their specific impacts on developmental outcomes. Further studies evaluating multi-year nutritional interventions are warranted to optimise human capital development across the life course.

Our findings aligned with previous studies show a positive association between low parental education and stunting [20,21]. Maternal education, in particular, her knowledge and health care-seeking behaviour, may contribute to better child nutrition. Conversely, low paternal education often reflects limited livelihood opportunities and poorer health behaviours, exacerbating nutritional risks for children [22].

The maternal low BMI was observed as a key predictor of post-discharge stunting in our study, and it is consistent with existing literature [23–25]. A study from Bangladesh conducted in 2011 reported that the risk of stunting was 21% among children born to underweight mothers compared to well-nourished mothers [26].

Our data implies that households with two or more under-five children, a higher risk of post-discharge stunting was higher. This may be due to the increased household disease burden, demonstrated by higher rotavirus detection in such settings. Additionally, food insecurity [27], restricting dietary adequacy, surging psychosocial stress on caregivers in larger families [28], *etc*. are responsible in this regard. These factors all together impair care, and dietary adequacy leads to poor linear growth.

Sociodemographic factors such as low parental education, maternal undernutrition, and larger numbers of young children in the household interact with broader socioeconomic disadvantages. Our multivariable approach aimed to estimate their independent associations with stunting, but we acknowledge that some shared variance may reflect underlying structural inequities.

Children admitted with some/severe dehydration due to diarrhoea showed significantly higher risk of stunting in 180 days post-discharge. Though the causality cannot be explained in our observational design, it is multifactorial [29]. Metabolic disturbances and nutrient losses in diarrhoea dysregulate anabolic growth pathways may impair growth in the long term [30,31]. Additionally, the resultant malnutrition and subsequent potential damage to gut epithelial cells impair nutrient assimilation for months after rehydration [32,33]. Socioeconomic stressors, recurrent infections, and epigenetic changes may also contribute to prolonged growth faltering [34–39].

Contrary to our expectations, exclusive breastfeeding was not found to be protective against post-discharge stunting after 180 days among children aged 2–23 months admitted with acute severe diarrhoea. The homogeneity in disease severity likely limits the ability to detect any potential protective effect of breastfeeding. The role of breastfeeding in mitigating longer-term growth faltering in this population with severe diarrhoea was not substantiated [40,41]. Furthermore, breastfeeding benefits may be affected by environments with persistent enteric infections and poor sanitation [41–44]. We acknowledge that exclusive breastfeeding was based on caregiver self-report, which may be prone to recall and social desirability biases, leading to potential misclassification. Furthermore, the low prevalence of exclusive breastfeeding in our cohort, fewer than 2%, limited the statistical power to detect any meaningful protective effects. These limitations contributed to the null association observed in our analysis. Future studies incorporating objective biomarkers of breastfeeding and larger sample sizes with greater variation in feeding practices may offer more definitive insights.

Local cultural, economic, and environmental factors are critical in shaping nutritional outcomes and must be considered when interpreting our findings. In low-resource settings like Bangladesh, food insecurity and poor health care access exacerbate the risk of stunting, particularly in rural or underserved communities [45,46]. Cultural practices, such as child-rearing practices and feeding behaviours, are often influenced by household income and maternal education [47]. As our study was conducted in diarrhoeal disease hospitals serving socioeconomically disadvantaged families, the association we observed may be strongly shaped by these broader social determinants [48]. Future studies should account for the complex interplay of these factors to refine intervention and ensure applicability across diverse populations [49].

Although we identified several key risk factors for post-discharge stunting, implementation of potential interventions in resource-constrained settings remains challenging due to limited health care infrastructure and socioeconomic constraints. To address these, we recommend community-based approaches, strengthening local health systems, and utilising mobile health technologies for follow-up. Enhancing maternal and child nutrition education, particularly in rural areas, can improve health-seeking behaviours and increase intervention effectiveness. Tailoring strategies to local contexts could help overcome barriers and improve stunting outcomes among under-2 children.

The main strength of our work is the representation of large data from the largest diarrhoeal disease hospital, which can make the study's findings relevant to the diarrhoeal population. We have analysed a large and compact data set with less missing data. Another important strength of the study is using predictive modelling, which helped to predict the status of stunting in the next six months of the children who were out of touch. This Predictive modelling approach is with low cost and less effort; we could assume the prevalence of stunting among this age group. Furthermore, the predictive model helps to address potential selection bias related to the initial disproportionate representation of children with severe stunting. By predicting outcomes for children who were not followed up, the model partially accounts for the potential differences between those who remained in the study and those who were not followed up, improving the overall representativeness of the findings.

A limitation of the study is that we have followed only the Bangladesh site data from selected hospitals, including children mostly with diarrhoea; other childhood illnesses could not be considered here in our data set. Moreover, while our study presents a predictive modelling approach to estimate post-discharge stunting, we acknowledged a few limitations. First, the model is based on data from two urban hospital sites in Bangladesh, limiting its generalisability to other contexts with differing socioeconomic or health care conditions. Additionally, the accuracy of the model is contingent on the quality and completeness of the data, and incomplete information may introduce bias. The model primarily uses baseline variables, which may not fully capture dynamic nutritional changes or environmental factors over time. Despite strong sensitivity and specificity, the model's real-world performance, particularly in resource-limited settings with limited follow-up, warrants further investigation. Future research should validate the model in diverse settings and refine it to incorporate additional risk factors and longitudinal data.

## CONCLUSIONS

Stunting is identified as one of the most serious health issues in Bangladesh. Identification of prevalence, risk factors, and forecasting of stunting will assist Bangladesh's government and developmental organisations in early planning and initiation and continue intervention to prevent future stunting load. Adult literacy/formal mass education programmes should be reinforced with knowledge of health education, child health, and nutrition. To maintain global nutrition momentum, a definite focus on nutrition investments, policies, and programmes on outcomes that truly matter will help to accelerate progress toward the well-being of children in disadvantaged communities.

## Additional material


Online Supplementary Document
