# Supplementary material for: Determinants of post-discharge stunting among diarrhoeal children aged 2–23 months in Bangladesh: findings from Antibiotics for Children with Severe Diarrhea (ABCD) trial
Source: J Glob Health. 2025 Jun 27;15:04185. doi: 10.7189/jogh.15.04185 (PMC12203627; doi:10.7189/jogh.15.04185)

Supplement to: **Nuzhat S, Das R, Kabir MF, Haque MA, Shahid ASMSB, Kamal M, Faruk MT, Ahmed T, Chisti MJ. Prediction of post-discharge stunting among diarrheal children. J Glob Health. 2025;15:04185.**

Table S1. Baseline characteristics of the total screened, enrolled, and not enrolled children aged 2–23 months in the Bangladesh site (ABCD trial) from 2017 to 2019

**Variables**	**Total screened*, n = 34 838 (%)**		**Enrolled*, n = 1431 (%)**		**Not enrolled*, n = 33 407 (%)**	
	**Not stunted, n = 29 244 (%)**	**Stunted, n = 5594 (%)**	**Not stunted, n = 842 (%)**	**Stunted, n = 589 (%)**	**Not stunted, n = 28 402 (%)**	**Stunted, n = 5005 (%)**
Gender (female)	12 605 (43.1)	1892 (33.8)	415 (49.3)	194 (32.9)	12 190 (42.9)	1698 (33.9)
Age in months						
2–11 months	19 473 (66.6)	3360 (60.1)	543 (64.5)	323 (54.8)	18 930 (66.7)	3037 (60.7)
12–23 months	9771 (33.4)	2234 (39.9)	299 (35.5)	266 (45.2)	9472 (33.3)	1968 (39.3)
Duration of diarrhea before hospitalization (days)†	2 (1,3)	2 (1,3)	2 (1,3)	2 (1,3)	2 (1,3)	2 (1,3)
Number of loose watery stools the child had in the past 24 hours†	10 (8,12)	10 (8,13)	10 (8,15)	10 (8,13)	10 (8,12)	10 (8,13)
Visible blood in stool	626 (2.1)	139 (2.5)	0 (0)	0 (0)	626 (2.2)	139 (2.8)
An antibiotic was taken at home within the last 14 days						
No	17 494 (59.8)	3726 (66.6)	842 (100)	589 (100)	16 652 (58.6)	3137 (62.7)
Yes	11 750 (40.2)	1868 (33.4)	0 (0)	0 (0)	11 750 (41.4)	1868 (37.3)
Breastfeeding status						
Exclusive breastfeeding	531(1.8)	100(1.8)	13(1.5)	6(1)	518(1.8)	94(1.9)
Mixed	27236(93.1)	5143(92)	783(93)	544(92.4)	26453(93.2)	4599(91.9)
Not breastfed	1472(5)	349(6.2)	46(5.5)	39(6.6)	1426(5)	310(6.2)
Dehydration on hospital admission						
No dehydration	28568(97.7)	5366(95.9)	561(66.6)	502(85.2)	28007(98.6)	4864(97.2)
Some/severe dehydration	676(2.3)	228(4.1)	281(33.4)	87(14.8)	395(1.4)	141(2.8)
Baseline anthropometry (mean ± SD)						
WAZ	-0.64±1.05	-2.24±0.87	-1.85±0.79	-2.81±0.57	-0.6±1.04	-2.17±0.88
LAZ	-0.6±0.87	-2.62±0.55	-0.98±0.7	-2.88±0.61	-0.59±0.88	-2.58±0.54
WHZ	-0.4±1.13	-0.99±1.13	-1.78±0.87	-1.6±0.92	-0.36±1.12	-0.92±1.13
MUAC (cm)						
≥11.5	28972(99.1)	5285(94.5)	827(98.2)	578(98.1)	28145(99.1)	4707(94)
<11.5	272(0.9)	309(5.5)	15(1.8)	11(1.9)	257(0.9)	298(6)
Maternal age†	25 (21.3,29)	24 (20.1,28)	24 (20.2,28)	23 (20,27.6)	25 (21.4,29)	24 (20.3,28)
Maternal BMI†	23.9 (21.1,27)	22.8 (19.8,25.9)	22 (19.5,25.4)	21.4 (18.9,24.8)	23.9 (21.2, 27.1)	22.9 (19.9,26.1)
Under five children in the household						

Only one child	24540(83.9)	4543(81.2)	715(84.9)	480(81.5)	23825(83.9)	4063(81.2)
Two children	4484(15.3)	987(17.6)	120(14.3)	101(17.1)	4364(15.4)	886(17.7)
≥ 3 children	220(0.8)	64(1.1)	7(0.8)	8(1.4)	213(0.7)	56(1.1)
Maternal education						
No education	2069(7.1)	800(14.3)	137(16.3)	135(23)	1932(6.8)	665(13.3)
Below primary	1994(6.8)	579(10.4)	107(12.7)	87(14.8)	1887(6.7)	492(9.9)
Primary and above	25142(86.1)	4197(75.3)	597(71)	364(62.1)	24545(86.5)	3833(76.8)
Paternal education						
No education	2603(8.9)	942(16.8)	164(19.5)	134(22.8)	2439(8.6)	808(16.1)
Below primary	1369(4.7)	445(8)	82(9.7)	64(10.9)	1287(4.5)	381(7.6)
Primary and above	25272(86.4)	4207(75.2)	596(70.8)	391(66.4)	24676(86.9)	3816(76.2)
Maternal decision making on child health care						
No	5502(18.8)	1058(18.9)	141(16.7)	120(20.4)	5361(18.9)	938(18.7)
Yes	23742(81.2)	4536(81.1)	701(83.3)	469(79.6)	23041(81.1)	4067(81.3)
Duration of hospital stay (hours)†	2.67 (2,10.3)	4.03 (2.2,17.9)	21.48 (10.8,29.1)	18.5 (8.3,25.3)	2.62 (1.9,8.7)	3.13 (2.1,15.7)
Outcome						
Alive	29233 (100)	5589 (99.9)	835 (99.2)	588 (99.8)	28398 (100)	5001 (99.9)
Death	11 (0)	5 (0.1)	7 (0.8)	1 (0.2)	4 (0)	4 (0.1)

BMI – body mass index, MUAC – mid upper arm circumference, LAZ – length-for-age, SD – standard deviation, WAZ – weight-for-age, WHZ – weight-for-height z scores.

*****Total screened: admitted to hospital for acute diarrhea; Enrolled: enrolled at ABCD trial; Not enrolled: excluded from the ABCD trial but admitted to the hospital for acute diarrhea.

†Median (interquartile range); Low Length/height-for-age (HAZ/LAZ<−2) or stunting; Breastfeeding status: mixed (both breastfed and complementary feed).

Figure S1. Directed Acyclic Graph (DAG)

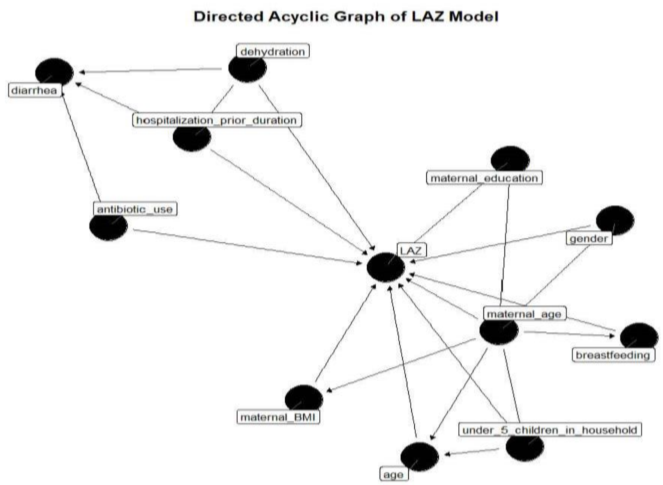

Supplement: Online Supplementary Document [file jogh-15-04185-s001.pdf]
